# Processing of a Zinc Leach Residue by a Non-Fossil Reductant

**DOI:** 10.1021/acsomega.3c00250

**Published:** 2023-06-05

**Authors:** Minna Rämä, Lassi Klemettinen, Marja Rinne, Pekka Taskinen, Radosław Markus Michallik, Justin Salminen, Ari Jokilaakso

**Affiliations:** † School of Chemical Engineering, Department of Chemical and Metallurgical Engineering, Aalto University, 02150 Espoo, Finland; ‡ Geological Survey of Finland, 02150 Espoo, Finland; § Boliden Kokkola Oy, 67101 Kokkola, Finland

## Abstract

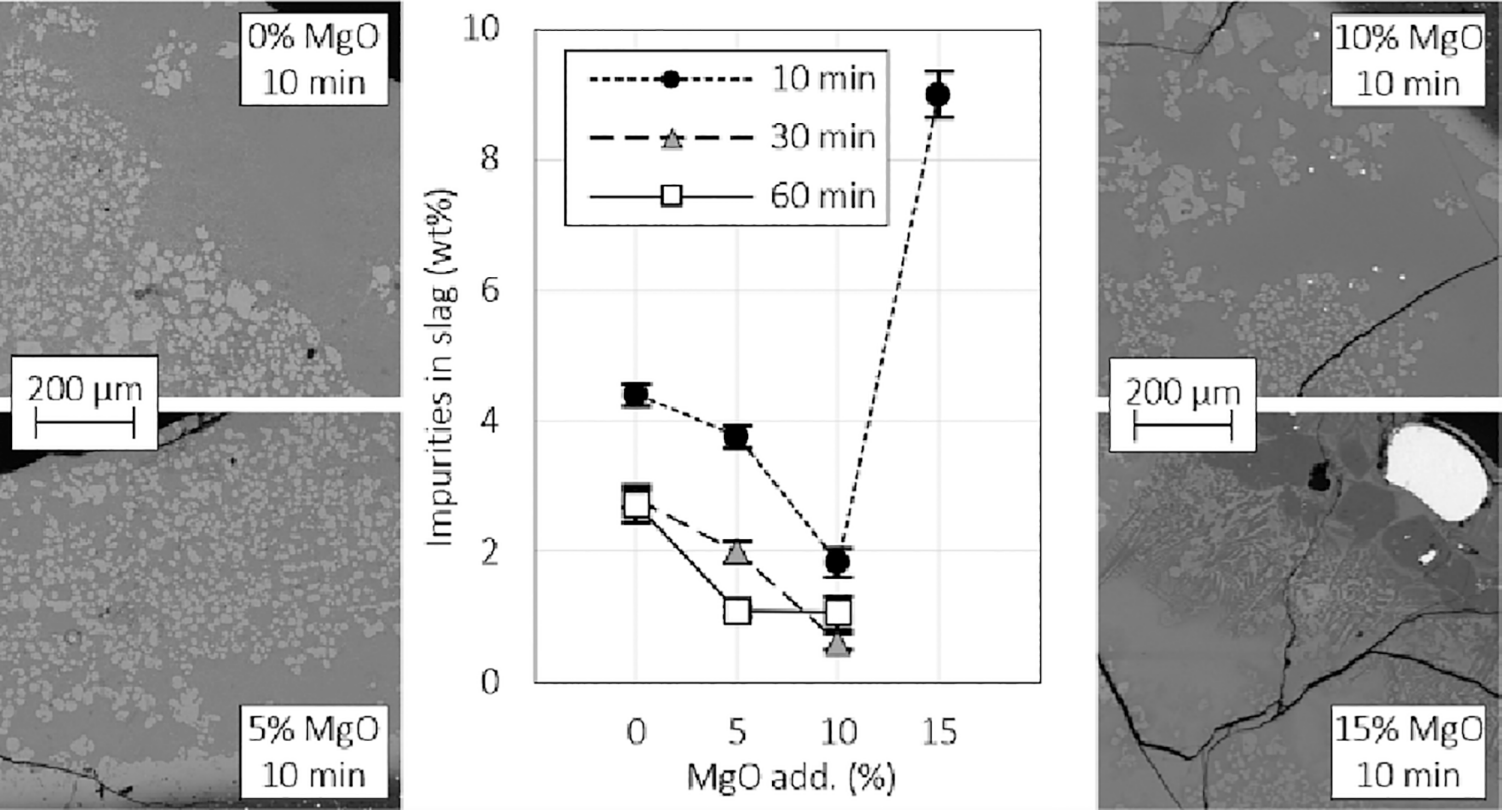

The suitability of a non-fossil reductant in high-temperature treatment of a zinc leach residue was studied in laboratory-scale experiments. The pyrometallurgical experiments carried out at temperatures of 1200–1350 °C consisted of melting the residue under an oxidizing atmosphere to produce an intermediate, desulfurized slag, which was further cleaned of metals such as Zn, Pb, Cu, and Ag, using renewable biochar as a reductant. The aim was to recover valuable metals and produce a clean, stable slag for use as construction material, for example. The first experiments indicated that biochar is a viable alternative to fossil-based metallurgical coke. The capabilities of biochar as a reductant were studied in more detail after optimizing the processing temperature at 1300 °C and modifying the experimental arrangement by adding rapid quenching of the sample (to a solid state in less than 5 s) to the procedure. Modifying the slag viscosity by adding 5–10 wt % MgO was found to enhance the slag cleaning significantly. With an addition of 10 wt % MgO, the target Zn concentration in slag (Zn < 1 wt %) was reached after as little as 10 min of reduction, and the Pb concentration was also decreased relatively close to the target value (Pb < 0.03 wt %). With an addition of 0–5 wt % MgO, the target Zn and Pb levels were not reached within 10 min, but with longer treatment times of 30–60 min, 5 wt % of MgO was enough to decrease the Zn content in slag sufficiently. The lowest Pb concentration achieved with an addition of 5 wt % MgO was 0.09 wt % after a 60 min reduction time.

## 1 Introduction

To avoid the worst possible consequences of global climate change, a significant decrease in the consumption of fossil carbon and the formation of CO_2_ emissions during metallurgical processes is required. Alternative non-fossil reductants, to replace the commonly used metallurgical coke, have started to attract widespread interest. Biochar as a reductant has already been studied in ferrous processes quite widely^1−3^ as well as in the processing of waste copper slag,^4,5^ and the results have indicated that biochar is a viable alternative in these metallurgical processes. The efficiency of zinc fuming with biochar in the pyrometallurgical treatment of the jarosite residue has been evaluated with a simple thermodynamic simulation, showing that only 10–30% more biochar is needed compared to the amount of coke required to achieve the typical recovery rates of zinc (> 90%).^6^ No previous experimental studies regarding the use of biochar in pyrometallurgical processing of jarosite residues were found; however, high-temperature reduction of the calcinated jarosite residue has been studied on a laboratory scale using hydrogen as a non-fossil reducing agent.^7^

Over the last few years, the annual total amount of zinc production worldwide has been about 13–14 Mt,^8^ of which the vast majority, about 85%, is produced by the roasting–leaching–electrowinning (RLE) process.^9^ To produce high-quality (99.995%) zinc by hydrometallurgical processing, the formation of an iron leach residue is inevitable, and iron removal from the leaching solution is needed before electrowinning.^10^ Most hydrometallurgical zinc processes utilize jarosite precipitation for controlling iron levels in the leaching stage. Jarosite has a chemical formula of XFe_3_(SO_4_)_2_(OH)_6_, where X represents either Ag^+^, H_3_O^+^, K^+^, Li^+^, Na^+^, NH_4_^+^, or ^1^/_2_Pb^2+^. Other possibilities are to precipitate iron as hematite (Fe_2_O_3_), goethite (FeO(OH)), or paragoethite (no unambiguous mineralogical characterization).^11,12^ The jarosite process is the most important iron precipitation route, and over the decades, this process has caused huge amounts of residue, which is to a large extent landfilled around zinc production sites. The estimated amount of jarosite currently produced worldwide is approximately 6.4 Mt per annum, and the amount is continually growing due to the increasing demand for zinc.^13^

Not only does zinc leach residue consists mainly of iron, impregnated lead sulfate, and zinc ferrite, but numerous other elements of the leaching solution are also co-precipitated in its complex matrix. There may be variation in the composition of the generated residue based on the process input, but the other elements can be, for example, unrecovered economically important metals (Cu, Ag, and Au), critical metals (In, Ga, Ge, and Sb), and elements of concern (Pb, As, Cd, and Hg), in addition to zinc and iron. Due to the hazardous nature of the jarosite residue, it is commonly stabilized before safe stockpiling.^14^ With current actions, the requirements of circular economy in the hydrometallurgical production of zinc are not being met as significant amounts of valuable metals, as well as potential raw materials, end up in landfills. As the demand for cement-based products is rapidly increasing due to global urbanization, it would be environmentally beneficial to utilize the treated residue for construction purposes.^15^ This would also help in dealing with the issue of limited availability of land for the stockpiling of hazardous residues. On the other hand, economic reasons, such as increasing landfilling costs in the future and economic benefits from recovering and recycling the valuable metals, are also arousing interest in processing of the residue.^10^

Several different methods for treating iron residues have been developed. Fundamentally, the options are either hydrometallurgical or pyrometallurgical processing or immobilization of the residue. In the review article by Hoeber and Steinlechner,^16^ all investigated strategies for iron residue processing have been thoroughly presented. The potential of pyrometallurgical treatment is shown by the fact that the only processes currently employed for treating iron residue on the industrial scale are pyrometallurgical. For example, Ausmelt Top Submerged Lance (TSL) technology is employed in Australia and South Korea, whereas the Waelz process is utilized in Brazil and China.^14,16^ In addition, a variety of pyrometallurgical treatments are being studied on a laboratory scale. The major advantages of pyrometallurgical treatment lie in producing an environmentally friendly and safely utilizable slag simultaneously with recovery of valuable metals, resulting in a minimum amount of waste. To improve the resource efficiency and the environmental impact of RLE processing, a simulation-based methodology has been used^17^ to determine the best option for integrating a pyrometallurgical flowsheet into a plant. Resource consumption, material recovery and losses, residue production, and social, environmental, and economic impacts, for example, were considered in the estimation. The results showed that treating the jarosite residue through oxidative melting and reduction stages seemed to be the best alternative.

The processing of jarosite residue in two-stage high-temperature (1250–1400 °C) treatment has proven to be a promising option for accomplishing both targets: a clean slag and recovery of valuable metals.^10,18,19^ First, the material is melted under oxidizing conditions to produce an intermediate slag consisting of metal oxides and to oxidize and remove sulfur. In addition, a metal oxide fume containing valuable metals, such as Zn, Pb, Ag, In, and Ge, is formed.^10^ The intermediate slag is further cleaned under reducing conditions, where the remaining valuable or harmful metals are removed either by volatilization or by forming a metal or speiss phase that can be separated based on density differences. Speiss is a metal alloy phase that forms under reducing conditions when As, Sb, and/or Sn are present. Basically, the speiss phases can be divided into two types: an iron arsenide speiss and a base metal speiss, the latter being a complex mixture of Cu, Ni, and Fe as arsenides and antimonides.^20^ For the iron residue treatment process, the formation of Cu-rich speiss would be desirable due to the fact that copper acts as an efficient collector for precious metals, such as silver.^21^ However, if metallic iron is present under highly reducing conditions, then Fe-As speiss with a high iron concentration starts to form as the stability of iron-arsenic alloys is higher than that of copper-arsenic alloys. With a higher degree of reduction, more metallic iron forms, resulting in less arsenic in the phase.^22,23^ Therefore, metallic iron in the system changes the nature of the speiss phase because copper dissolves in the phase only if there is enough arsenic present.^21^ As a result, copper and valuable metals are not recovered in the speiss, hindering their overall recovery in the process. It is therefore extremely important to avoid the formation of a large amount of metallic iron during the reduction step of the treatment. Ideally, the non-hazardous and clean slag that is produced could be used, e.g., for construction purposes. The volatilized valuable metals can be recovered from the fume by a hydrometallurgical process.^10^ Promising results, with recovery rates of 90% Zn, 99% Pb, 75–85% Ag, 60–70% In, and 80–90% Ge, have been obtained by treating the jarosite and sulfur residue in a two-stage ArcFume plasma process.^10^

The main target of the current study was to assess the feasibility of a bio-based reducing agent, biochar, in the reduction stage of the high-temperature processing of the zinc leach residue to produce a clean slag and to recover valuable metals.

## 2 Experimental Section

### 2.1 General Experimental Approach

The pyrometallurgical treatment of the iron-rich zinc leach residue was investigated at 1200–1350 °C on a laboratory scale. The aim was to convert the iron residue into a clean slag suitable for construction purposes, for example, and to recover valuable metals such as Zn, Pb, Cu, and Ag. During the first experimental series (ES1), the primary objective was to study the suitability of a bio-based reductant instead of a fossil one to achieve the goals of the treatment. The objective of the second experimental series (ES2) was to study whether the process could be improved by lowering the viscosity of the slag. The thermal treatment consisted of two stages. First, the pre-treated material was melted under oxidizing conditions to produce an oxide melt with S < 1 wt %. The solid reductant was added on top of the desulfurized material (after cooling) for the reduction stage, where the target was to clean the slag of valuable and harmful metals, aiming at a slag composition with Zn < 1 wt % and Pb < 0.03 wt %.^10^ To produce a slag that is suitable for construction purposes, i.e., classified as inert waste, leaching of certain elements from the slag has to be below the strict limits (Zn < 4 mg/kg, Pb < 0.5 mg/kg) set by the standard leaching test.^24^ Based on previously conducted leaching tests, the abovementioned targets for Zn and Pb have been found to be sufficient.^10^ The intention was to either volatilize Zn, Pb, Cu, and Ag to the gas phase or to form a metal alloy or a Cu-rich speiss phase to be separated from the clean slag based on differences in their densities.

### 2.2 Materials and Pre-Treatment

The sample material used originated from the leaching stage of an industrial hydrometallurgical zinc production process (RLE). Its chemical composition was 3.1 wt % Pb, 0.9 wt % Zn, 16.0 wt % Fe, 5.0 wt % Ca, 1.4 wt % Na, 2.4 wt % Si, and 26.5 wt % S. The detailed initial composition was omitted, but the residue also contained traces of other metals, such as Cu, Al, Ag, As, Sb, Hg, Cd, Ge, and In.

The material was dried at 100 °C for 24 h in an air atmosphere in a muffle furnace (Memmert GmbH + Co. KG, Germany). Prior to the experiments, the dried and ground material was thermally decomposed at 700 °C for 60 min to release elemental sulfur, sulfates, hydroxyl groups, and residual moisture.^25^ Assuming that the majority of the iron residue is sodium jarosite, the total reaction during the decomposition can be described using eq 1.^25,26^
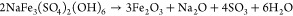
1

For the pre-treatment, a dense MgO crucible (SC10030, 20/75 mm ID/H, Tateho Ozark Technical Ceramics, USA) containing the sample material was gradually lifted using an alumina tube into the hot zone of a vertical high-temperature furnace (LTF 16/-/450, maximum temperature 1600 °C, Lenton, UK), where it was kept in a flowing air atmosphere (65 mL/min, pressurized air). The furnace was equipped with an impervious pure alumina working tube (Frialit AL23, Friatec AG, Germany, 45/38 mm OD/ID). The temperature of the hot zone was monitored with an alumina sheath-covered, calibrated Pt/Pt10Rh thermocouple (Johnson Matthey, UK, uncertainty ±3 °C) located next to the sample, connected to multimeters (2000 and 2010, Keithley, USA). After the set time, to avoid cracking of the crucible or the working tube, the crucible was slowly lowered (during 10 min) and cooled down to room temperature. Several treatments were conducted, and the batches were mixed together to homogenize the material for the subsequent experiments. Rotameters (Kytola Instruments, Finland, accuracy ±5% FS) were used for regulating the gas flow rates of air, O_2_, and Ar during the pre-treatments and further oxidation–reduction experiments. The off-gas cleaning system and a more detailed description of the equipment and pre-treatment procedure have been presented in earlier publications.^18,19^

After pre-treatment, the Fe/SiO_2_ ratio of the material was adjusted to 1.86 (w/w) to obtain the correct orthosilicate slag composition for the oxidation–reduction experiments. This composition has been shown to be effective in minimizing losses of valuable metals in slag.^27^ The ratio was changed by adding the required amount of SiO_2_ (sand, 274739, Sigma-Aldrich, USA). For the samples with an addition of MgO in the ES2 experiments, the desired amount of MgO (99.95%, Alfa Aesar, USA) was also added at this point. The material was mixed in a mortar to ensure homogeneity. A hydraulic press (15-834, Biltema, Sweden) was employed for pressing the sample material into pellets of approximately 4 or 2 g, depending on the experimental setup used.

The biochar used in the reduction step was produced from Finnish PEFC-certified spruce (Carbons Finland Oy, Finland) at 600 °C with a 45 min residence time. The low ash content (3.1 wt %) of the dried biochar combined with its relatively high fixed carbon content (C-fix = 90 wt %) made it suitable for metallurgical use. The metallurgical coke (size fraction 8–20 mm), obtained from a Finnish company, was ground into powder so as to be suitable for the experiments. Its ash content was 11–12 wt %, thus noticeably higher than that of biochar, and the C-fix was lower (86 wt %). The content of volatiles in the biochar (4.0 wt %) was higher than in the coke (0.5–1.0 wt %), but it was assumed that the volatile components of both reductants would be gasified when the sample was lifted into the hot zone of the furnace.

### 2.3 Smelting Experiments

#### 2.3.1 General Targets and Experimental Parameters

During the first stage, the target was to decrease the sulfur concentration to S < 1 wt %. The formation of base metal sulfides, such as FeS, ZnS, PbS, or Cu_2_S, was also prevented by desulfurization as the presence of a liquid matte would drastically hamper the removal of zinc from the melt. In addition, most of the zinc and lead in the material were expected to be volatilized as ZnO and PbO, respectively, and an intermediate slag formed when all remaining elements were oxidized. During the subsequent reduction stage, the target was to further decrease the zinc concentration in the slag to below 1 wt % and the lead concentration to below 0.03 wt %.

The experimental study was divided into two sections. The first experimental series (ES1) included 10 experiments with larger samples (2 × 4 g pellets) that were slowly lifted up into the hot zone and, after the set time, slowly lowered from there and cooled down. To obtain a more accurate understanding of the microstructures of the molten samples, the sample size was reduced to 2 × 2 g pellets for the second series (ES2), which included 29 experiments. This enabled the sample to be hung from the top of the furnace, making it possible to quench it in ice water after the set time. Thus, the molten state microstructures could be preserved for subsequent characterization. The experimental parameters for both experimental series are shown in Table 1.

**Table 1:** Experimental Parameters for Experimental Series 1 and 2

info	temperature (°C)	MgO add. (wt %)	O_2_ flow rate (mL/min)	reduction time (min)	reductant amount	reductant type
ES1 (large sample, slow cooling)	1200^a^	0	65			
	1200	0	65	20/30/40	2 × stoich.	biochar 100%
				20/30/40		coke 100%
				20/30/40		coke 50%, biochar 50%
ES2 (small sample, rapid quenching)	1200^a^/1300^a^/1350^a^	0	65			
	1300^a^	0/5/10	32/65			
	1300	0	32/65	10/30/60	0.5 × stoich.	biochar 100%
	1300	5	32/65	10/30/60	0.5 × stoich.	biochar 100%
	1300	10	32/65	10/30/60	0.5 × stoich.	biochar 100%
	1300	15	32/65	10	0.5 × stoich.	biochar 100%

a Refers to the reference experiments including only the oxidation stage of the treatment.

#### 2.3.2 Experimental Series 1

For ES1, the same equipment and procedures were used as for the pre-treatments. A schematic and a more detailed description of the experimental arrangement have been presented in earlier studies.^18,19^ In ES1, samples of 2 × 4 g pellets in larger MgO crucibles (SC10012, 20/32 mm ID/H, Tateho Ozark, USA) were used. The experimental temperature was 1200 °C to follow the process at lower temperatures than earlier. This would decrease the energy consumption of the process. The material was already successfully melted at this temperature due to fluxing with SiO_2_. During the oxidation treatment, the O_2_ (99.98%, Linde, Finland) flow into the furnace was 65 mL/min and treatment time was 60 min. A gas lance was applied to enhance the oxygen flow on top of the sample as it had been proven to have a positive impact on the sulfur content achieved.^19^ After the set time, the crucible was lowered from the hot zone and cooled to room temperature. One reference experiment including only oxidation treatment was conducted, and for the other samples, the oxidation step was followed by a reduction treatment.

Based on the remaining sample mass after oxidation, the stoichiometric amount of carbon needed for the reduction was calculated. Twice that amount of carbon was added on top of the sample to ensure the availability of the reductant. To examine the impact of the non-fossil reductant, three different cases were tested. One set of experiments was conducted using biochar as a reductant, another was conducted with metallurgical coke, and for the third set, equal amounts by weight of biochar and coke were mixed together. For the reduction step, three reduction times of 20, 30, or 40 min and an inert atmosphere with a 300 mL/min argon flow (99.999%, Linde, Finland) were used. Various treatment times for the reduction stage were tested because the lead concentration of the slag in the preliminary tests with a gaseous reductant rapidly decreased between 20 and 40 min. The gas lance was excluded from the arrangement to ensure an inert atmosphere throughout the working tube.

#### 2.3.3 Experimental Series 2

**Figure 1:**
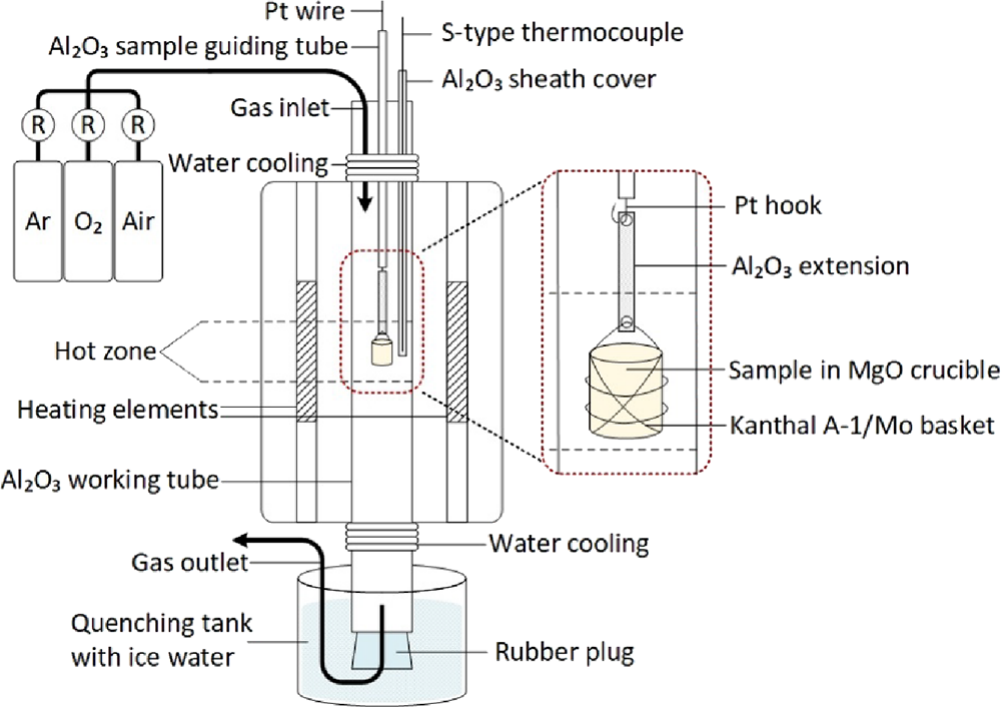
Schematic of the experimental setup. R = rotameter.

The experimental arrangement of ES2 was modified for quenching the sample rapidly instead of slow cooling, as shown in Figure 1. Since the crucibles used in the previous experiments were too heavy to hang from the top of the furnace, smaller MgO crucibles (SC07510, 13/25 mm ID/H, Tateho Ozark, USA) were used, reducing the sample size to 2 × 2 g pellets. Platinum wire (Ø 0.50 mm, Johnson Matthey, USA) inside an Al_2_O_3_ guiding tube was used for pulling the sample up into the hot zone. To ensure that the sample would not fall during the experiments despite the high temperatures and rather high total weight, the hook of the Pt wire was placed above the hot zone. This was achieved by connecting a 20 cm Al_2_O_3_ extension tube with drilled holes to the Pt wire on one end, while the other end was connected to the basket holding the crucible and the sample. The Pt wire was pulled up so that the extension stick reached the guiding tube as the sample had then reached the hot zone. For the oxidation treatments, the basket was made of Kanthal A-1 wire (Ø 0.50 mm), and for the reduction treatments, it was made of Mo wire (Ø 0.50 mm, Plansee, Austria). Due to the modifications made to the furnace, it was not possible to use a gas injection lance during the second experimental series. This was assumed to have a slight effect on the efficiency of the oxidation treatment.

For the oxidation treatment, the sample was lifted up in an air atmosphere, which was changed to O_2_ (32 or 65 mL/min) after the hot zone was reached. The reductions were conducted under flowing Ar (300 mL/min).

At the set time, the rubber plug was removed from the bottom end of the working tube, and a quenching tank with ice water was placed under the furnace so that the ice water surface was above the bottom end of the work tube, sealing air from the work tube during the quenching. After the set time, by sharply pulling the Pt wire, the hook holding the extension stick opened, and the sample dropped down into the ice water and was rapidly quenched. The sample was quenched after both stages to preserve the high-temperature microstructures. The biochar was added on top of the sample before proceeding to the reduction stage.

Due to the findings of the previous studies and observations made during ES1, it was assumed that spinels^19^ or solid iron oxide particles in the slag matrix hindered the formation of a bigger, uniform, metal or speiss phase during the reduction treatment as the metal or speiss droplets seemed to be attaching to them. A notable fraction of magnetite in the slag matrix was observed after the first quenched oxidation treatment conducted at 1200 °C. A test was made to see if the amount of magnetite could be reduced by raising the treatment temperature. Temperatures of 1300 and 1350 °C were tested for the 60 min oxidation treatment with an O_2_ flow of 65 mL/min. Based on the results, 1300 °C was chosen for the further experiments, including both the oxidation and reduction stages.

The reductant amount was determined based on the sample mass after the oxidation stage. In ES2, however, the reductant amount was only 0.5 times the stoichiometric need. The amount of reductant was decreased from the ES1 experiments as it was observed that large Fe-As speiss phases formed locally, on top of the melt, when the FeO in the slag was locally over-reduced to metallic iron. For ES2, treatment times of 10, 30, and 60 min were chosen for testing in the reduction stage to obtain a more comprehensive picture of the kinetics compared with the previous experiments.

As the slag cleaning was not sufficient and solid iron oxide particles were still present despite increasing the experimental temperature, the viscosity of the melt was decreased by adding MgO to the initial material, thus enhancing slag cleaning. MgO additions of 5 and 10 wt % were introduced to the pre-treated and fluxed material, and the results were compared to those without any added MgO. Furthermore, the effect of a smaller oxygen amount during oxidation was tested to investigate whether the same results could be obtained using less oxygen. Therefore, the reduction experiments were conducted with two intermediate slags that were produced differently as the oxidation stage was conducted using either a 32 or 65 mL/min O_2_ flow. Based on the results obtained, an addition of 15 wt % MgO was also tested with both slags. These 15 wt % MgO tests were done with a 10 min reduction time as the results showed that the biggest changes in the Pb, Zn, and As content in the slag could be observed during the first 10 min. Reference experiments including only the oxidation step were conducted with all combinations of parameters.

**Figure 2:**
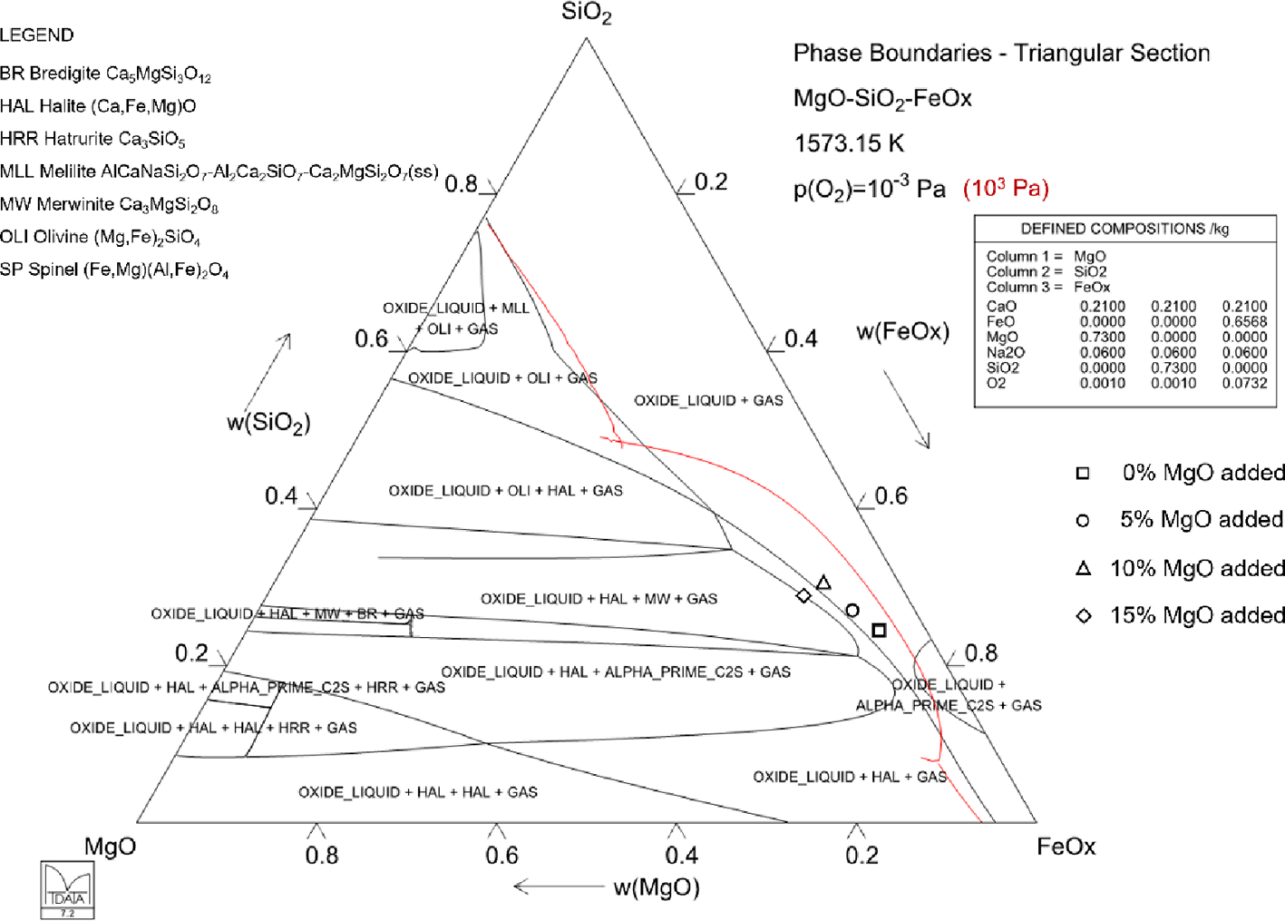
Quasi-ternary isothermal section of the MgO-SiO_2_-FeOx system at 1300 °C. Four slag compositions with varying MgO additions, based on experimental results, are superimposed. The phase diagram contains constant CaO (21 wt %) and Na_2_O (6 wt %) concentrations. The black lines are calculated for 10^–3^ Pa oxygen partial pressure (reduction stage) and the red lines for 10^3^ Pa (oxidation stage, liquidus line only).

During the oxidation stage at 1200–1350 °C, pure molten iron silicate slag is not stable. However, the high concentrations of other oxides present in the zinc leach residue (CaO, Na_2_O, K_2_O, ZnO, and PbO) decrease the liquidus temperature of the molten slag, enabling the formation of a molten intermediate slag, thus increasing the sulfur removal rate from the material. Figure 2 presents a quasi-ternary isothermal section of the FeOx-SiO_2_-MgO system at 1300 °C with constant CaO and Na_2_O concentrations (21 and 6 wt %, respectively), calculated with MTDATA using the MTOX database.^28^ The black lines represent the phase boundaries during the reduction stage (10^–3^ Pa oxygen partial pressure was chosen), and the red line depicts the molten slag-halite saturation boundary at a higher oxygen partial pressure (10^3^ Pa), chosen to represent the oxidation stage. According to the phase diagram, the molten slag area increases significantly when the oxygen partial pressure decreases.

The phase diagram indicates how the saturation boundary moves along with the prevailing oxygen partial pressure, i.e., the degree of reduction. The red contour shows the saturation boundary of the liquid oxide at *p*(O_2_) = 10^3^ Pa, which at low silica concentrations is at halite (wüstite, FeO) saturation and at very high silica concentrations at olivine ((Fe,Mg)_2_SiO_4_) saturation. When the prevailing oxygen partial pressure is decreased during the reduction process, ferric oxide of the slag is converted to ferrous oxide, according to eq 2.
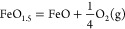
2

This continuous adjustment in the operation point along with the degree of reduction also shifts the saturation boundary of the solid phases toward higher MgO concentrations, as shown by the black phase boundary calculated at *p*(O_2_) = 10^–3^ Pa. The reduced state also modifies the stable phases so that solid wüstite (halite) is the primary phase at low silica concentrations up to about 29 wt % of SiO_2_, and olivine predominates above that. It should be noted that in the SiO_2_ corner of the phase diagram, the slag actually contains 73 wt % of SiO_2_ with 21 wt % CaO and 6 wt % Na_2_O.

Four experimental points from this work (ES2, 60 min reduction for 0–10 wt % MgO addition and 10 min reduction for 15 wt % MgO) were superimposed onto the graph. With 0–10 wt % MgO added to the system, a solid iron oxide phase was detected. Therefore, these experimental points should be located on the molten slag-halite saturation boundary. The small discrepancies between the experimental points and the phase diagram calculation can be attributed to other oxides present in the system at low concentrations (< 5 wt % total), the variation between the actual and estimated oxygen partial pressure, or the need to optimize the database used for the calculation. In the case of the addition of 15 wt % MgO, another solid phase, merwinite (Ca_3_MgSi_2_O_8_), was detected. Therefore, this experimental point should be located on the merwinite saturation boundary, which it is.

### 2.4 Sample Characterization

The samples were mounted in epoxy resin and prepared for SEM-EDS and EPMA analyses by traditional metallographic methods including grinding and polishing. The finished samples were washed in ethanol for 10 min using an ultrasonic cleaner (M3, FinnSonic, Finland). The cross sections were carbon-coated with a vacuum evaporator (JEOL IB-29510VET, Jeol Ltd., Japan) to ensure sufficient electrical conductivity on the sample surface during the analysis. The microstructures of the samples were examined with scanning electron microscopy (SEM) (Mira3 SEM, Tescan, Czech Republic), and the phase compositions were analyzed with energy-dispersive spectrometry (EDS) (UltraDry 30 mm^2^ EDS, Thermo Fisher Scientific, USA). An acceleration voltage of 15 kV and beam current of approximately 11 nA on the sample surface were used during the analyses. The elemental compositions of the phases were quantified using the standard materials presented in Table 2.

**Table 2:** Analyzed Elements, X-ray Lines, Standard Materials, and Diffraction Crystals Used in This Work

	X-ray lines and standards		diffraction crystals
element	EDS	EPMA	EPMA
O	Kα, diopside	Kα, aluminum oxide	PC0
Na	Kα, tugtupite	Kα, tugtupite	PC0
Si	Kα, quartz	Kα, quartz	TAP
Al	Kα, aluminum	Kα, almandine	TAP
Mg	Kα, magnesium	Kα, diopside	TAP
Ca	Kα, fluorite	Kα, diopside	LPET
K	Kα, sanidine	Kα, sanidine	LPET
S	Kα, marcasite	Kα, pentlandite	LPET
Mn	Kα, manganese	Kα, rhodonite	LLIF
Fe	Kα, hematite	Kα, hematite	LLIF
Co	Kα, cobalt	Kα, cobalt	LLIF
As	Lα, cobaltite	Kβ, GaAs	LLIF
Zn	Kα, zinc	Kα, sphalerite	LLIF
Ni	Kα, nickel	Kα, nickel	LLIF
Cu	Kα, copper	Kα, copper	LLIF
Ag	Lα, silver	Lα, silver	LPET
Sb	Lα, antimony	Lα, SbTe	LPET
Ba	Lα, barite	Lα, barite	LPET
Pb	Mα, lead	Mα, galena	LPET

As rapid quenching was utilized for the ES2 samples, relatively homogeneous slag and iron oxide phases (as well as a merwinite phase in samples with 15 wt % MgO addition) were obtained. These phases were analyzed using an electron microprobe (EPMA) at the Geological Survey of Finland. The microprobe used was an SX-100 (Cameca SAS, France) equipped with five wavelength-dispersive spectrometers (WDS). The accelerating voltage was 20 kV, and the beam current was 40 nA. For the iron oxide and merwinite phases, a focused beam was used, and for slag, a 20 μm defocused beam was chosen. Eight points were analyzed in each phase, and the analytical results were corrected using the PAP online correction program.^29^ The analyzed elements, X-ray lines, and standard materials used are collected in Table 2. The detection limits and dwell times can be found in the Supporting Information, Table S1.

The bulk chemical compositions of both the dried and the pre-treated material were determined. The sample size of the first experimental series allowed their bulk chemical composition determination as well, but it was not possible for the second experimental series due to the small sample size. For the chemical analysis of the first series, it should be noted that it contains all the formed phases as they were not mechanically separated from each other. An inductively coupled plasma–optical emission spectroscopy (ICP-OES) device (iCAP 6000, Thermo Fisher Scientific, USA) was used, and microwave-assisted digestion of the samples was done with HNO_3_, HCl, and HBF, using a MARS 6 device (CEM Corporation, USA).

## 3 Results and Discussion

### 3.1 Pre-Treatment

In earlier studies, pre-treatment was shorter, only 15 min,^18,19^ after which the decrease in the sulfur concentration was around 31%. Since the objective of the pre-treatment is sulfur removal, to simplify the following oxidation stage, an even lower sulfur content would be desirable. With the 60 min treatment, a decrease of around 38% was reached; thus, this treatment time was used. During pre-treatment, most sulfur was released right after the beginning of the treatment as elemental sulfur accumulation in the off-gas cleaning system was clearly visible. Therefore, increasing the treatment time may not have been beneficial for the process from the resource effectiveness perspective, even though some more sulfur was removed. Photos of the iron residue before and after pre-treatment are shown in the Supporting Information, Figure S1.

### 3.2 Results of ES1

#### 3.2.1 Oxidation Stage of ES1

To reach the target sulfur concentration (S < 1 wt %) after desulfurization in oxidizing conditions, approximately 96% removal of the original sulfur was needed in total. The reference experiment included only oxidation at 1200 °C, and the target sulfur level was reached. The sample size of the first experimental series allowed the conduction of bulk chemical analysis with ICP-OES in addition to the SEM-EDS analysis. The results obtained with the two methods were in good agreement with each other, with a sulfur concentration of the intermediate slag at 0.4 wt %, based on both the SEM-EDS and ICP-OES results.

The SEM BSE (backscattered electron) microstructure images showed that, under an oxidizing atmosphere, molten slag and iron oxide particles of Ø 10–50 μm were formed. The iron oxides spread in the slag matrix rather evenly throughout the sample, and they were mostly angular-shaped, presumably indicating that they were not molten. The formation of iron oxides during the treatment differed from our previous study,^19^ where Mg-rich spinels were observed instead. According to the current findings, spinels could be destabilized or the dissolution of magnesium in the phase reduced by changing the Fe/SiO_2_ ratio through silica fluxing. The EDS results showed that the slag consisted mainly of iron oxides (Fe = 17.2 wt %), along with CaO (22.2 wt %) and SiO_2_ (31.4 wt %). A smaller share of the slag was made up of other oxides, such as MgO, Na_2_O, Al_2_O_3_, MnO, K_2_O, and BaO. Slag basicity calculations are presented in the Supporting Information, Tables S2 and S3. The fraction of lead in the slag after the oxidation treatment was high (8.7 wt %), even though it was expected to be largely removed to the gas phase as PbO. This clearly indicated the demand for further slag cleaning under a reducing atmosphere to reach the target lead concentration. However, the zinc concentration was already below the target, with the slag containing only 0.7 wt % of zinc. Other elements remaining in the slag after the oxidation stage in low concentrations were As, Ni, Sb, Co, and Ag.

The notable fraction of distinct iron oxide particles in the sample needs to be considered when evaluating the composition of the whole sample as it is not possible to separate them from the slag. The EDS results showed that they consisted mainly of iron (60.3 wt %) and oxygen (26.8 wt %), but other elements, such as zinc (5.4 wt %) and magnesium (2.3 wt %), were also detected. Based on the Fe/O atomic ratio, it was concluded that the iron oxides were mainly magnetite (Fe_3_O_4_). Their exact share in the slag matrix cannot be determined based on only a few cross sections of the sample as the microstructure showed variation throughout the sample. The high zinc concentration, however, supports the need for further treatment of the melt under reducing conditions. The zinc and lead concentrations in the whole sample, including both the slag and the magnetite particles, based on the ICP-OES chemical analysis, were 1.7 and 4.6 wt %, respectively.

#### 3.2.2 Reduction Stage of ES1

The 20, 30, and 40 min reductions were performed at 1200 °C, with either metallurgical coke, biochar, or a 50:50 mixture of these as a reducing agent. Twice the amount of carbon needed for the reduction, calculated based on stoichiometry, was used to ensure the sufficiency of the reductant for the treatment. The reduction reactions considered in carbon amount calculations are presented in the Supporting Information.

**Figure 3:**
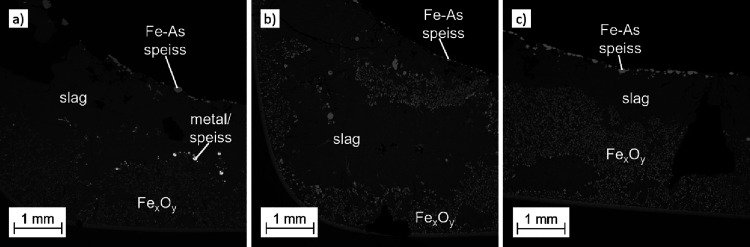
SEM BSE microstructure images of the samples after a 40 min reduction at 1200 °C using (a) metallurgical coke, (b) biochar, and (c) 50:50 mixture of coke and biochar as a reductant.

Figure 3 shows the SEM BSE microstructure images of the samples after a 40 min reduction with metallurgical coke, biochar, and the 50:50 mixture. The prevailing phases in all samples after the reduction treatment were molten slag (dark gray matrix), iron oxides (light gray areas), iron arsenide speiss (lightest gray areas at the top surface), and Pb-rich alloy/speiss droplets (in Figure 3a, the bright spots). The microstructures shown in Figure 3 were developed already after a 20 min treatment, and no significant differences were observed in the microstructures between the samples treated for 20, 30, and 40 min with different reductants (Supporting Information, Figure S3). The melt was clearly divided into different areas. The iron oxide particles were mostly concentrated at the bottom part of the crucible, whereas the cleaner slag without the oxides was usually observed at the top. This indicated that the iron oxide particles in the upper part of the melt, where the reductant was also placed, had already reduced to FeO and dissolved into the slag and that the reduction proceeded slowly from top to bottom.

The iron oxide particles, Ø 10–50 μm in size, were round, indicating that they were molten, unlike in the oxidation step (Supporting Information, Figure S2). The size of the particles remained the same regardless of the time or the reductant used. The main elements were the same as after the oxidation treatment, but more iron (71.9 wt %) and less oxygen (19.7 wt %) were detected. In all of the reduction experiments, the zinc concentration of the particles had decreased to below 1.9 wt %. The Fe/O atomic ratio suggests that the magnetite particles that were seen in the microstructure after the oxidation treatment were reduced to ferrous oxide during the reduction treatment. However, the reduction had not been sufficient because plenty of solid particles were still present in the slag matrix and had not dissolved in the slag as FeO. In a previous study, where the jarosite residue was treated using hydrogen as a reductant, the reduction had clearly proceeded further because the slag matrix was mainly free of iron oxides after only a 15 min reduction.^7^ The presence of incompletely reduced iron oxides in the slag matrix is considered detrimental because they increase the viscosity of the slag so that metal droplets tend to attach to them, possibly preventing the formation of a larger metal phase or hindering their volatilization to the gas phase. In addition, their presence causes the non-homogeneity of the bulk material and the target Fe/SiO_2_ ratio in slag was not obtained because some iron was deported in iron oxide particles. In ES1, the samples were slowly cooled to room temperature after the experiments. Therefore, it is also unclear how much of the observed microstructure was already present at the experimental temperature. Possible reactions during the iron oxide reduction are presented in the Supporting Information.

During the reduction stage, the Fe^3+^ of the intermediate slag should be reduced to Fe^2+^ (FeO). The reductant was placed on top of the sample, and the experimental arrangement did not allow any stirring of the melt. Therefore, the mass transfer between the melt and the reductant was poor, leading to reduction of FeO further to metallic iron in the upper part of the sample while the reduction at the bottom of the crucible was incomplete. In the presence of metallic iron, Fe-As speiss is formed under strongly reducing conditions due to the high stability of Fe-As alloys.^22,23^ Thus, a layer of Fe-As speiss phases (Ø 50–250 μm) with a high iron concentration was detected on the top surface, where the melt had evidently come into good contact with the reductant, and metallic iron formation had occurred. In addition, a speiss phase forming on the surface of the melt presumably hinders the release of volatiles, such as Pb and Zn, to the gas phase. Based on the EDS analysis, the Fe-As speiss phases were not homogeneous, but had two different compositions, of which the more Fe-containing ones contained approximately 84.6 wt % of Fe and 10.2 wt % of As. The other Fe-As speiss phases consisted of approximately 57.7 wt % of Fe and 31.8 wt % of As. With a higher degree of reduction, more metallic iron was formed, leading to an increase in the concentration of Fe in the phase.^22,23^

This speiss differs from the one reported in the earlier experimental study,^19^ where Cu-Sb speiss had formed instead of Fe-As speiss. In the current study, only very few Cu-rich speiss droplets were detected, with most of the speiss phases formed during the reduction stage being Fe-As-based. This can be explained by the formation of metallic iron and the high stability of arsenic-iron alloys compared to arsenic-copper alloys. It was observed for all of the reductants used that, by increasing the treatment time, the fraction of iron in the Fe-As speiss increased, whereas that of arsenic decreased. In addition, this may have been affected by the difference in the copper content of the initial sample material, considering that in the previous studies, the intermediate slag after the oxidation step contained approximately 0.39 wt % of Cu, whereas the present material contained only 0.09 wt % of Cu. However, the formation of a Cu-rich speiss would have contributed to the recovery of valuable and harmful metals that are not volatilized from the melt due to the ability of copper to absorb those metals.

Lead as such is not formally a part of speiss; however, when a speiss phase is formed, lead often accompanies it due to its geochemical association with many base metal ores and low melting point.^20^ This was also observed in the current study, where for some samples, up to 100 × 250 μm in size, Pb-rich (75.4–93.6 wt %) phases were detected at the top of the melt together with Fe-As speiss. Smaller Pb-rich droplets (79.3–90.9 wt %) of around 5–10 μm in diameter were detected mainly in the lower part of the crucible, attached to the iron oxide particles. These droplets were almost invariably associated with small Fe-As speiss phases as well. With a longer reduction time, only a small increase in the average size of the Pb-rich droplets was observed, but some individual droplets with diameters of up to 100 μm were detected. Using different reductants or treatment times did not affect the composition of the droplets. In addition to lead, they contained small amounts of other metals, such as Sb, Ag, Fe, As, and Cu. Because the metals did not coalesce to form a single, larger metal droplet at the bottom of the crucible, their separation from the slag would not have been possible.

**Figure 4:**
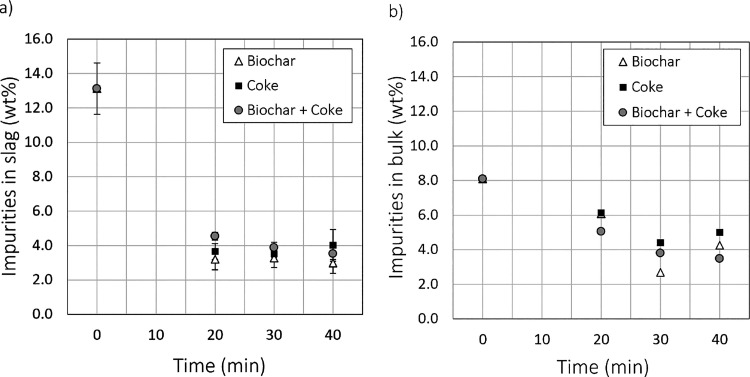
Total amount of impurities (Ni, Co, Zn, Pb, Ag, Cu, S, Sb, and As) in wt % in (a) slag (EDS analysis) and (b) bulk material (ICP-OES analysis) after 20, 30, and 40 min of reduction with different reductants.

As can be seen in the microstructures (Figure 3), the samples were quite heterogeneous, having numerous iron oxide particles and metal/speiss droplets in the slag matrix. Thus, the chemical analysis of the whole sample gives an uncertain result of the slag composition. However, it allows estimation of the effect of different reductants and treatment times on the evaporation of certain elements from the bulk material. A more exact slag composition can be determined by EDS analysis, but in reality, the iron oxide particles and metal/speiss droplets could not be separated from the slag matrix to obtain a clean slag without them. Based on the EDS analysis, the main components of the slag were FeO (30.6–34.9 wt %), SiO_2_ (29.0–32.7 wt %), and CaO (20.2–25.7 wt %), with oxides of Na, Mg, Al, K, and Mn also present. Additionally, the slag contained some impurities, such as Ni, Co, Zn, Pb, Ag, Cu, S, and possibly traces of As and Sb, that were not successfully removed from the slag. The efficiency of the different reductants and the kinetics of the reduction stage were evaluated by the proportion of the total amount of impurities in the slag after the treatment. Figure 4a shows the total amount of impurities (Ni, Co, Zn, Pb, Ag, Cu, S, Sb, and As) in the slag based on the EDS results as a function of reduction time for the three different reductants. Correspondingly, the total concentration of impurities in the bulk material, containing iron oxide particles and metal/speiss droplets in addition to slag, based on the ICP-OES chemical analysis, is shown in Figure 4b.

Based on the EDS analysis, the slag after the oxidation step contained approximately 13.1 wt % of impurities in total. Already after 20 min of reduction treatment, the total amount of impurities in slag had decreased drastically, and only a little progress was seen with longer treatment times. After each treatment time, a lower total impurity content in the slag was reached with biochar rather than with coke. The chemical analysis of the bulk material also showed that, with biochar as a reductant, the impurities were more efficiently volatilized than with coke as a reductant. These results are in agreement with a previous study comparing nickel slag cleaning efficiency using coke and biochar as reductants.^30^ Based on the chemical analysis, the removal of impurities from the bulk continued steadily until 30 min with all reductants. It is somewhat unclear why the total impurity concentration in the bulk material increased between 30 and 40 min, but it may be related to sampling: larger Pb-rich or speiss droplets may have ended up in the material selected for analysis. An additional reason may be the decreased slag volume due to the reduction of iron. Based on the results, the increase was due to the increased Pb concentration, which supports the sampling-related conclusion. Errors such as this clearly illustrate the drawbacks of total chemical analysis for heterogeneous samples.

The removal of especially zinc and lead from the melt is crucial for producing a clean slag. It was seen from the EDS results that the target for the zinc concentration in slag was reached already after the oxidation step, and a further decrease was observed in the reduction stage. The lowest zinc levels in slag were obtained with biochar as a reductant, and after a 40 min treatment, there was 0.2 wt % of Zn in slag, whereas with coke or the 50:50 mixture of coke and biochar, 0.4 wt % of Zn in slag was detected. Based on the EDS analyses, using biochar as a reductant also resulted in lower Pb levels in the slag, compared to the treatments with coke or the 50:50 mixture. The ICP-OES analyses supported the EDS results, and the lowest achieved levels of Zn and Pb were obtained with biochar. After a 40 min treatment with biochar, 0.8 wt % of Zn and 1.5 wt % of Pb in the bulk were achieved.

### 3.3 Results of ES2

#### 3.3.1 Treatment Temperature Selection

As the Pb-rich droplets accompanying the Fe-As speiss seemed to be attaching to the iron oxide particles during the reduction treatment in the ES1 experiments, the objective was to investigate whether the formation of any distinct iron oxide phase could be avoided, thus producing a more homogeneous slag. The oxidation treatment of the first experimental series was repeated with rapid quenching of the sample instead of slow cooling. Two higher temperatures, 1300 and 1350 °C, were tested as well, again with rapid quenching of the sample.

The SEM BSE microstructure images showed that, after rapid quenching of the sample oxidized at 1200 °C, there was a considerable fraction of small (Ø 10–30 μm) iron oxide particles, mostly angular-shaped, throughout the whole cross section. Only very small areas of homogeneous slag without these particles were visible. The microstructures also showed some larger (Ø 50–100 μm), angular-shaped iron oxide particles with high (around 7.4 wt %) Pb concentrations close to the top surface of the sample. The angular shape suggests that they were in solid form at the treatment temperature. The microstructure of the sample oxidized at 1300 °C still contained iron oxide particles; however, there were fewer of them, and they were larger (Ø 30–100 μm) and rounder in shape. In addition, the fraction of homogeneous slag was larger. Most importantly, solid iron oxides with a high Pb concentration were not found at the increased temperature. No significant differences were seen between the samples treated at 1300 and 1350 °C in the EDS analyses nor in the microstructures.

The EDS analyses showed that the sulfur level had decreased below the target (S < 1 wt %) in all treatments at the tested temperatures. In slag, the total amounts of trace elements (mostly Pb, As, Zn, Ni, S, and Co) were 11.8, 8.7, and 8.5 wt % after the 60 min oxidation at 1200, 1300, and 1350 °C, respectively. Thus, the increased temperature seemed to promote the cleaning of slag already during the oxidation stage. The temperature increase boosted especially the removal of Pb as its concentrations in slag were 6.8, 4.4, and 3.8 wt %, after oxidation at 1200, 1300, and 1350 °C, respectively. No clear effect of the increased treatment temperature on the Zn concentration in slag was observed. Based on the obvious enhancements in the microstructures and slag compositions between the samples treated at 1200 and 1300 °C, and only minor changes when the temperature was increased to 1350 °C, further experiments were conducted at 1300 °C, and other practices were investigated to intensify the treatment.

#### 3.3.2 Oxidation Stage of ES2

During the differently conducted oxidation treatments, the phases formed were molten slag and solid iron oxide particles that were evenly distributed in the slag matrix. Their shape was mostly rounded, implying that they were molten at 1300 °C. No distinct differences were seen in the microstructures despite the different treatment parameters, including the MgO additions and oxygen flow rates during the treatment. SEM BSE micrographs of samples after the oxidation stage are shown in the Supporting Information, Figure S4.

The EPMA analyses showed that the slag consisted mostly of SiO_2_ (24.9–32.9 wt %), CaO (19.5–24.3 wt %), and iron oxides (Fe = 9.3–23.7 wt %). The rest of the oxides were mainly MgO, Na_2_O, Al_2_O_3_, and BaO. When the addition of MgO was increased from 0 to 5 and 10 wt %, naturally, there was more MgO in the slag as well, but the proportion of SiO_2_ and CaO also increased, whereas the proportion of iron oxides decreased. Changing the O_2_ flow rate did not distinctly affect the slag composition. Each sample reached the target sulfur level in the slag. In addition, no sulfur was found in the iron oxides. The Zn concentration in slag was 0.9–1.1 wt % and Pb > 3.1 wt % in all samples, with no clear correlation with time or MgO addition. Even though the Zn concentration in slag was close to the target value (Zn < 1 wt %), further treatment was needed, especially to obtain Pb levels closer to the target (Pb < 0.03 wt %). In all of the samples, other impurities were also present in the slag, for example, As (1.4–1.9 wt %) and Sb (0.1–0.2 wt %).

The iron oxide particles (Ø 30–100 μm) consisted mainly of iron (53.0–58.4 wt %) and oxygen (28.9–31.3 wt %) but also of other elements, such as magnesium (6.0–11.0 wt %) and zinc (2.2–4.5 wt %). Based on the Fe/O atomic ratio, the particles consisted mostly of magnetite (Fe_3_O_4_), which is similar to the results after ES1 oxidation treatment. The O_2_ flow rate used during the treatment did not seem to affect the phase composition. Naturally, with increasing MgO addition, from 0 to 5 wt % and further to 10 wt %, it could clearly be seen how an increasing amount of iron in the iron oxide phase was replaced by magnesium. The highest zinc levels in the phases were detected in the samples without any addition of MgO.

#### 3.3.3 Reduction Stage of ES2

**Figure 5:**
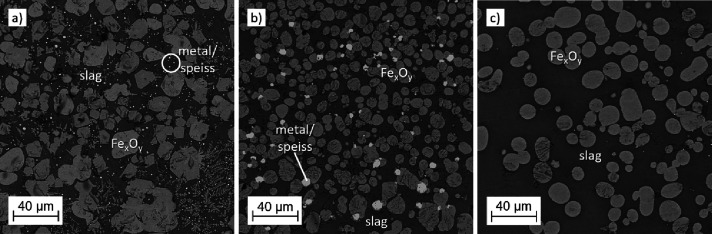
SEM BSE microstructure images of samples after reduction treatment at 1300 °C with (a) no MgO addition, 10 min, (b) 10 wt % MgO addition, 10 min, and (c) 10 wt % MgO addition, 60 min. The intermediate slag used had been produced with 32 mL/min O_2_ flow during oxidation.

The phases formed during the reduction treatment were molten slag, iron oxide particles (Ø 5–50 μm), and metal droplets of varying size and composition. These droplets consisted mainly of a Pb-rich phase together with an Fe-As speiss. Some larger (Ø 30–100 μm) Fe-As speiss droplets were detected in samples with the higher (10 or 15 wt %) MgO additions after 10 and 30 min treatments, but not anymore after 60 min. Figure 5 shows different types of microstructures formed during the reduction treatment of the intermediate slag produced with 32 mL/min O_2_ flow, focusing on the areas with more iron oxides. Figure 5a,b represents samples treated for 10 min. In Figure 5a, no MgO was added to the initial sample material, and for Figure 5b, 10 wt % of MgO was added. The microstructure shown in Figure 5c is from a sample with an addition of 10 wt % MgO, but with a longer, 60 min, treatment time. The dark gray matrix in the microstructures is slag, and the lighter gray phases are iron oxides. Pb-rich metal and Fe-As speiss droplets are the bright spots that are very small in Figure 5a and larger in size and attached to the iron oxides in Figure 5b. Most iron oxide particles were located in the lower part of the melt. There were no significant differences between the microstructures when the intermediate slag used had been treated with either 32 or 65 mL/min O_2_ flow during oxidation. The dendrites in the slag shown in Figure 5a were most likely formed during quenching.

With no addition of MgO, after reductions of 10 and 30 min, there were lots of very small, unsettled metal/speiss droplets in the slag matrix in the area where the iron oxide particles were located, as shown in Figure 5a. After a 60 min reduction, however, they were not detected anymore. The samples with 5 wt % MgO addition showed very small droplets in the slag matrix after a 10 min treatment. After a 30 min treatment, only a few droplets attached to iron oxides were detected. Furthermore, no droplets were detected after 60 min. After 10 and 30 min treatments with an addition of 10 wt % MgO, droplets attached to iron oxides were detected, as shown in Figure 5b. Figure 5c shows that after a 60 min treatment with an addition of 10 wt % MgO, there were practically no unsettled droplets present in the slag anymore. Thus, increasing the amount of MgO added promoted the coalescence of very small metal/speiss droplets in the slag matrix into larger droplets already with a shorter treatment time. These droplets were often attached to the iron oxides after shorter reduction times.

Based on the EPMA analyses, the slag consisted mainly of FeO (16.9–38.6 wt %), SiO_2_ (21.8–31.8 wt %), and CaO (17.3–24.7 wt %) after each reduction experiment. Smaller fractions of other oxides, such as MgO, Na_2_O, BaO, and Al_2_O_3_, as well as impurities, such as Zn, Pb, Ni, and Co, were also detected. The variations in slag composition were mainly due to the additions of MgO, as was seen already after the oxidation stage.

**Figure 6:**
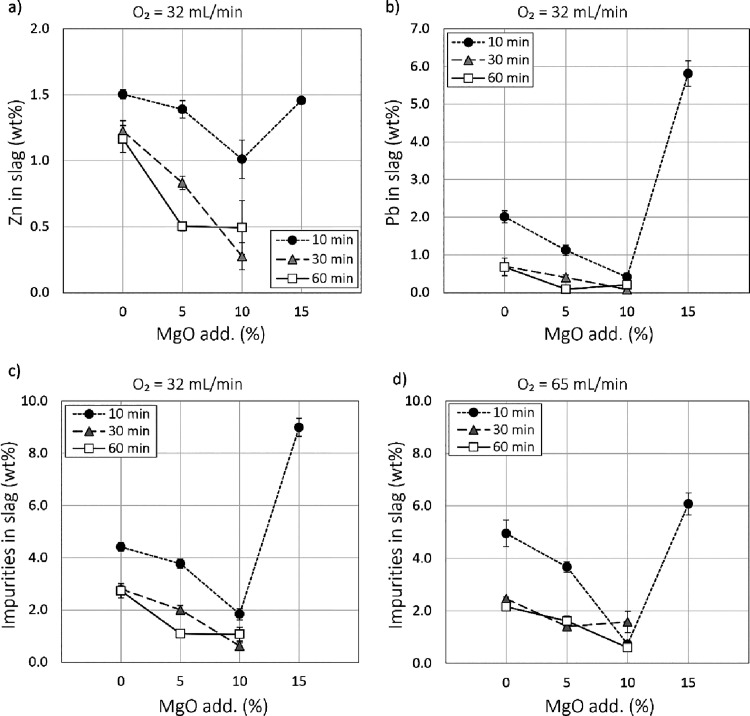
Effect of MgO addition on (a) Zn and (b) Pb concentrations (intermediate slag produced with 32 mL/min O_2_ flow), and on the total amount of impurities (Ni, Co, Zn, Pb, Ag, Cu, S, Sb, and As), for the two intermediate slags; (c) 32 mL/min and (d) 65 mL/min O_2_ flow in slag (wt %) after 10, 30, and 60 min reduction times based on EPMA analyses.

The removal of especially zinc and lead was enhanced by the increased amount of MgO in the initial material. Figure 6a and Figure 6b show the Zn and Pb concentrations in slag, respectively (intermediate slag produced with 32 mL/min O_2_ flow), after the 10, 30, and 60 min treatment as a function of MgO addition. With no MgO addition, the target Zn level (Zn < 1 wt %) in the final slag was not reached even after the longest treatment time (60 min), but with 10 wt % MgO addition, the target was reached after only 10 min. The same effect was seen again for lead, where 2.0 wt % of Pb was detected in both (32 and 65 mL/min O_2_ flow) intermediate slags without any MgO after a 10 min treatment. Adding 5 wt % MgO resulted in 1.1 and 1.3 wt % of Pb after 10 min, respectively, for intermediate slags produced with 32 or 65 mL/min O_2_ flow. The addition of 10 wt % MgO promoted the removal even more, and after a 10 min reduction, 0.41 and 0.08 wt % of Pb were correspondingly detected for the two intermediate slags.

Figure 6c,d shows the effect of the addition of MgO on slag cleaning of all impurities (Ni, Co, Zn, Pb, Ag, Cu, S, Sb, and As) after the 10, 30, and 60 min reduction treatments. The results are shown separately for intermediate slags produced with either 32 or 65 mL/min O_2_ flow during the oxidation stage. Both the MgO addition and the treatment time had a clear impact on the removal of impurities from slag. The effect of adding MgO was most pronounced in the 10 min treatments. With the addition of 10 wt % MgO, after only 10 min, the total fractions of impurities in slag were 1.8 and 0.7 wt % for the two differently produced intermediate slags. As shown in Figure 6c,d, these levels were not reached even after a 60 min treatment without MgO. The oxygen flow used during the oxidation stage did not have a systematic impact on the results after reduction treatment.

Since the effect of the addition of MgO was most pronounced after 10 min treatments, additional tests with 15 wt % MgO addition were conducted for a 10 min reduction only to see if the cleaning of slag could be improved even more. As seen in Figure 6a,b, increasing the MgO addition to 15 wt % resulted in insufficient removal of Zn and Pb from the slag. Further, Figure 6c,d shows how the 15 wt % MgO addition decreased the overall slag cleaning of all impurities. The reason for the poorer results after 15 wt % MgO addition may be attributed to the increased viscosity of the slag with higher MgO concentrations due to the formation of another solid phase, merwinite (see Figure 2).

The solid iron oxide particles are distributed throughout the slag matrix and cannot be separated from it, and therefore must be considered as part of the slag. Thus, it is essential to analyze their composition as well to determine whether there are considerable amounts of harmful or valuable elements. The iron oxide phases consisted mainly of iron (49.7–67.0 wt %), oxygen (24.8–30.2 wt %), and magnesium (2.0–20.2 wt %), with the different added amounts of MgO being the main cause for the large variation. Based on the atomic ratios, the magnetite particles that were present in the slag matrix after the oxidation treatment had started reducing during the reduction stage, similarly to the ES1 reduction stage. However, the reduction had been incomplete because not all magnetite was reduced to FeO and thus dissolved in the slag. The addition of increasing amounts of MgO to the initial sample material led to a phase composition where more iron was replaced by magnesium. A small amount of zinc (0.7–2.3 wt %) was also detected in the solid iron oxides. Both the increasing treatment time and the increasing amount of MgO added seemed to have a slight effect on reducing the amount of Zn in the phase. The lowest concentrations of Zn in the iron oxides were detected in samples with 10 wt % MgO addition. Similarly to the ES1 experiments, the presence of the incompletely reduced iron oxide particles in the slag matrix was assumed to hinder the volatilization of Zn and Pb as well as metal/speiss coalescence and settling processes by increasing the slag viscosity and trapping metal/speiss droplets.

**Figure 7:**
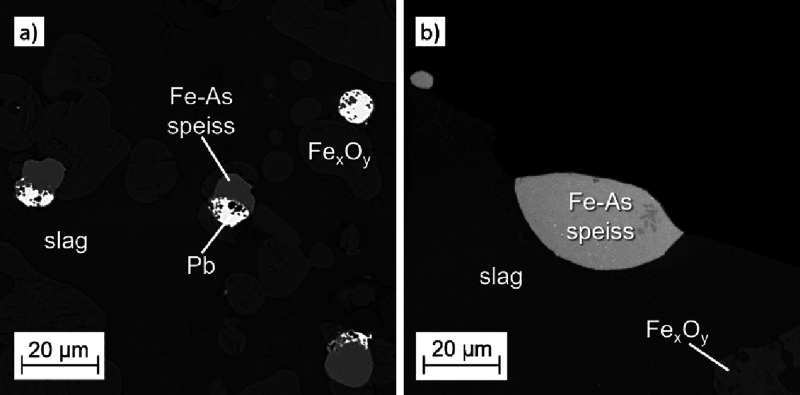
Metal/speiss phases formed during the reduction stage; (a) Pb-rich droplets accompanied by Fe-As speiss after a 30 min treatment of a sample with 10 wt % MgO addition (32 mL/min O_2_ during oxidation); (b) Fe-As speiss formed at the top of the melt after a 10 min treatment of a sample with 10 wt % MgO addition (65 mL/min O_2_ during oxidation).

The very small bright droplets seen in the microstructure (see Figure 5a) after the 10 and 30 min experiments with no MgO addition were < 2 μm in diameter, and based on their appearance, they were considered to be metal/speiss phases. The small size, as well as the large number of individual droplets, made further phase composition analysis challenging. Thus, it was also problematic to assess whether the compositions of the droplets changed between the experiments. However, based on the qualitative EDS results, it can be suggested that the droplets were composed mainly of lead and also contained small amounts of iron, arsenic, copper, antimony, and silver in varying proportions. Figure 7a shows a close-up of the microstructure after a 30 min reduction treatment when an addition of 10 wt % MgO was made to the initial material, and the intermediate slag produced with 32 mL/min O_2_ flow was used. In samples with more MgO, small metal/speiss droplets coalesced together, forming droplets of approximately 2–20 μm in diameter, attached to iron oxide particles. SEM-EDS analyses showed that the droplets often consisted of two phases, Pb-rich metal alloy and Fe-As speiss, as shown in Figure 7a. The Pb-rich phase consisted mainly of lead (73.9–90.2 wt %) with smaller fractions of Sb, As, and Fe. The Fe-As speiss consisted mainly of Fe (59.6–63.4 wt %) and As (25.0–32.8 wt %).

For samples with an addition of 10 or 15 wt % MgO, after 10 and 30 min treatments, some larger Fe-As phases, commonly 30–100 μm in diameter, were detected at the top surface of the melt. Based on EDS analysis, they had compositions of 55.4–66.7 wt % of Fe and 26.8–35.3 wt % of As. In addition, a total of 4.6–8.1 wt % of Al, Co, Ni, Cu, Sb, and Ag combined was detected. Figure 7b shows this type of phase located at the surface of the melt after a 10 min reduction treatment for a sample with 10 wt % MgO addition. Compared to the reduction experiments in ES1, the overall formation of these phases was distinctly lower, indicating that the smaller amount of reductant added on top of the sample had helped prevent their development. In addition, the iron concentration in the Fe-As phase was not as high as in the ES1 samples and apparently the arsenic concentration had been sufficient to allow some copper dissolution in the phase as well.

Since the formation of a Cu-rich speiss would promote collection of the valuable metals in one phase, it was essential to examine whether these phases were generated, and which experimental parameters were favorable. Some very small Cu-rich speiss droplets were detected, but their existence seemed to be coincidental rather than due to changing any experimental parameter. Cu-rich speiss also contained Ni, Sb, As, Pb, and Ag. Because the formation of Fe-As speiss with a high iron concentration was significantly lower than in ES1, it is likely that it did not hinder the formation of Cu-rich speiss. It is, therefore, clear that the reason was the very low copper concentration in the starting material.

## 4 Summary and Conclusions

The aim was to investigate the potential of biochar as a reductant in the high-temperature treatment of the zinc leaching iron residue, with the objective of removing harmful and valuable metals through the reduction of the oxidized and desulfurized intermediate slag. The Fe/SiO_2_ ratio of the residue was adjusted to the common field of iron silicate slags. Two series of experiments were carried out, the first of which (ES1) investigated the ability of biochar to act as a reductant, compared to the metallurgical coke commonly used in metallurgical processes. In the second series (ES2), the viscosity of slag was modified by adding MgO (5, 10, or 15 wt %) to the initial material, and the oxygen flow rate in the oxidation stage was varied to improve the performance of the reduction treatment in terms of slag purity, by promoting the deportment of volatiles into the gas phase, or impurity accumulation in speiss.

The oxidation stages of both experimental series successfully achieved the targeted sulfur level (S < 1 wt %) in slag. Zinc and lead were partially removed during the oxidation stage by releasing to the gas phase as ZnO and PbO, respectively. The results showed, however, the necessity for further treatment of the melt under reducing conditions to achieve the targeted levels of Zn (< 1 wt %) and Pb (< 0.03 wt %). In particular, the concentration of lead was high after the oxidation step. Further, although the analyses showed that the zinc concentration in the molten slag was at the target (ES1) or close to it (ES2), high zinc levels were observed in the magnetite particles within the slag matrix. The effect of temperature on the slag cleaning during oxidation was evaluated by comparing the ES1 oxidation treatment with the ES2 oxidation treatment conducted with an O_2_ flow of 65 mL/min and without the addition of MgO. Based on the results, increasing the temperature from 1200 to 1300 °C promoted the removal of lead from slag, but the same improvement was not observed for zinc.

The formation of iron oxide phases during the oxidation step was not observed in our earlier study,^19^ where Mg-rich spinel formation occurred during the treatment. The SiO_2_ fluxing in the current study either destabilized the spinels or decreased the rate of magnesium dissolution in the solid phase. The iron oxide phases consisted mostly of magnetite (Fe_3_O_4_). Studies on lead blast furnace slags have shown that, in particular, the presence of solid magnetite in slag greatly increases the viscosity, causing metallic lead entrapment in slag.^31^ Thus, the presence of the magnetite particles in the samples of the current study hindered the lead removal from the slag.

In the ES1 experiments, biochar and coke were used as reductants. The results showed a clear trend in enhanced slag cleaning with increasing treatment time for both reductants, but systemically better results were obtained with biochar. The high reactivity of biochar^32^ was concluded to be advantageous, and thus, its suitability in reduction treatment of the iron residue was confirmed. However, the processing parameters, particularly temperature, used during preparation of the biochar, i.e., the pyrolysis, influence its properties. Therefore, in future studies, we will investigate which biochar properties are most favorable for processing iron residues. The results of the ES2 reduction treatments showed the effect of adding MgO to the initial material, thus lowering the slag viscosity and resulting in noticeably more efficient slag cleaning, evidently due to increased activity coefficients of the less basic oxides.^33^ Adding 10 wt % of MgO resulted in a smaller fraction of impurities in slag after 10 min than after a 30–60 min treatment without addition of MgO. However, further experiments with addition of 15 wt % MgO showed the limitations of enhancing the slag cleaning by adding MgO due to its limited solubility, resulting in the formation of another solid phase, merwinite. The possibility of decreasing the reaction time needed for such pyrometallurgical treatment at high temperatures brings significant improvement in processing capacity.

Twice the stoichiometrically needed amount of reductant was used in ES1. This resulted in the local reduction of FeO to metallic iron, leading to the formation of a layer of Fe-As speiss on top of the slag. It was considered important to avoid this so as not to lose any valuable metals in the slag or by dissolution in the Fe-As speiss, from where valuable metal recovery would be more difficult than from a Cu-rich speiss. Therefore, the amount of reductant was decreased for ES2, and the formation of Fe-As speiss was successfully lowered. However, no larger Cu-rich speiss droplets were formed in either of the experimental series of this work. The formation of Cu-rich speiss can be promoted by modifying the initial composition of the iron residue.

It was concluded that the ferric oxides in the iron oxide particles started reducing to ferrous oxides during the reduction stage. Especially on top of the sample, where the contact with the reductant was better, the ferric oxides reduced completely and dissolved in the slag. It was expected that, with longer treating times, the reduction of magnetite particles would proceed further, resulting in lower viscosity. This was observed in ES2, where longer reduction times resulted in metal droplets attached to iron oxides being removed from the slag matrix. It is possible that the longest (40 min) treatment time in ES1 was not sufficient for this as metal droplets were still visible. The additions of MgO in ES2 clearly enhanced the coalescence of the metal droplets, even with shorter treatment times, through the decreased viscosity of the slag and increased activity coefficients of less basic oxides. The effect of temperature on slag purification during the reduction stage was evaluated by comparing certain ES1 and ES2 cases (biochar as a reductant, 30 min treatment time, 65 mL/min O_2_ flow, no MgO addition, and 1200 or 1300 °C). Increasing the temperature from 1200 to 1300 °C did not have much impact on the efficiency of slag cleaning as it did not result in lower levels of Zn and Pb in the slag. However, even though the temperature in ES2 was higher, the amount of the reductant used was only a quarter of that used in ES1, suggesting that the reductant amount used in ES2 may have been too low.

An appropriate amount of reductant as well as stirring of the melt, to considerably improve the contact of the reductant with the melt, will promote the reduction of solid magnetite particles to wüstite, and thus their dissolution in slag. In future studies, the lack of stirring could be compensated by mixing the reductant properly with the intermediate slag, instead of placing it on top of the sample, before lifting it up into the hot zone for reduction. This would presumably result in better contact between the reductant and the slag, leading to a more homogeneous final microstructure without locally reduced or otherwise differently reacted areas. It was found that twice the stoichiometric amount of reductant led to an unfavorable result with the formation of metallic iron. Thus, the reductant amount could be adjusted to find the optimal amount between the two options investigated in this work. By preventing the formation of Fe-As speiss phases containing large amounts of iron on top of the melt and possibly adding a small amount of copper-containing material, the conditions for the desired formation of Cu-rich speiss would be more favorable.

## Supporting Information Available

EPMA detection limits and dwell times for ES2 (Table S1); photos of the iron residue before and after the pre-treatment (Figure S1); SEM BSE micrographs after the oxidation stage of ES1 and after a 40 min reduction with biochar in ES1 (Figure S2); SEM BSE micrographs of 20, 30, and 40 min reductions with a 50:50 mixture of biochar and coke in ES1 (Figure S3); SEM BSE micrographs after the oxidation stage of ES1 and ES2 with 5 wt % MgO and 32 or 65 mL/min O_2_ (Figure S4); slag basicity after ES1 and ES2 experiments (Tables S2 and S3); possible reactions and reaction enthalpies during the reduction of iron oxides (eqs S1–S7); equations used for calculating the amount of reductants needed (eqs S8–S11) (PDF)
