# Supplementary material for: Processing of a Zinc Leach Residue by a Non-Fossil Reductant
Source: ACS Omega. 2023 Jun 5;8(24):21450–63. doi: 10.1021/acsomega.3c00250 (PMC10286245; doi:10.1021/acsomega.3c00250)
Supplement: Supplementary file 1 — ao3c00250_si_001.pdf [file ao3c00250_si_001.pdf]

Processing of a Zinc Leach Residue by a Non-Fossil Reductant

Minna Rämä [1,*], Lassi Klemettinen [1,*], Marja Rinne [1], Pekka Taskinen [1], Radosław Markus Michallik [2], Justin Salminen [3], Ari Jokilaakso [1]

[1] Aalto University, School of Chemical Engineering, Department of Chemical and Metallurgical Engineering, 02150 Espoo (Finland)
[2] Geological Survey of Finland, 02150 Espoo (Finland)
[3] Boliden Kokkola Oy, 67101 Kokkola (Finland)
* corresponding author

Supporting Information

Table S1. EPMA detection limits and dwell times for experimental series 2.

element	detection limits (ppm)		dwell times (s)	
	iron oxide	slag	peak	total background
O	1291	1647	30	30
Na	302	240	20	20
Si	142	146	30	30
Al	156	133	30	30
Mg	194	175	30	30
Ca	85	107	20	20
K	87	85	20	20
S	107	103	20	20
Mn	180	183	20	20
Fe	197	183	20	20
Co	266	290	10	10
As	2213	2145	90	90
Zn	319	303	20	20
Ni	225	215	20	20
Cu	256	245	20	20
Ag	325	312	20	20
Sb	283	368	20	20
Ba	277	297	20	20
Pb	352	357	60	60

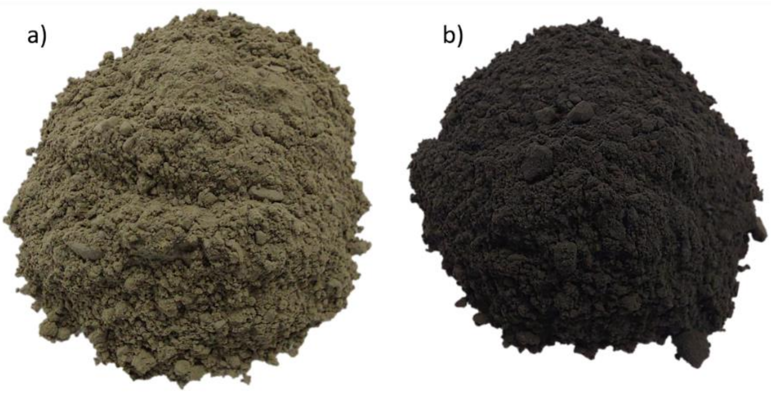

Figure S1. Photos of the iron residue (a) before and (b) after the pre-treatment.

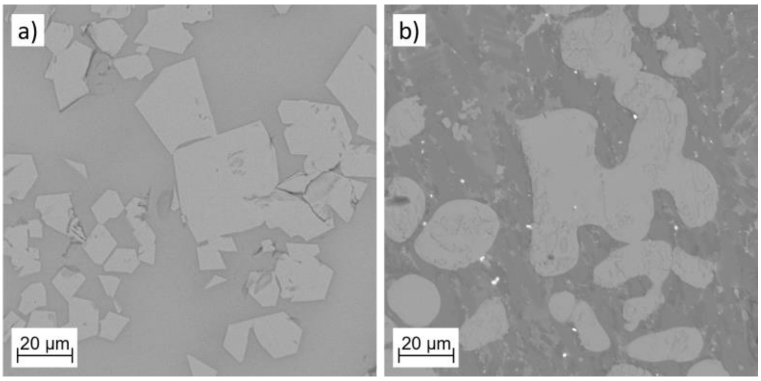

Figure S2. SEM BSE micrographs (a) after the oxidation stage of ES1 and (b) after a 40 min reduction with biochar (ES1); comparison of the shapes of iron oxides.

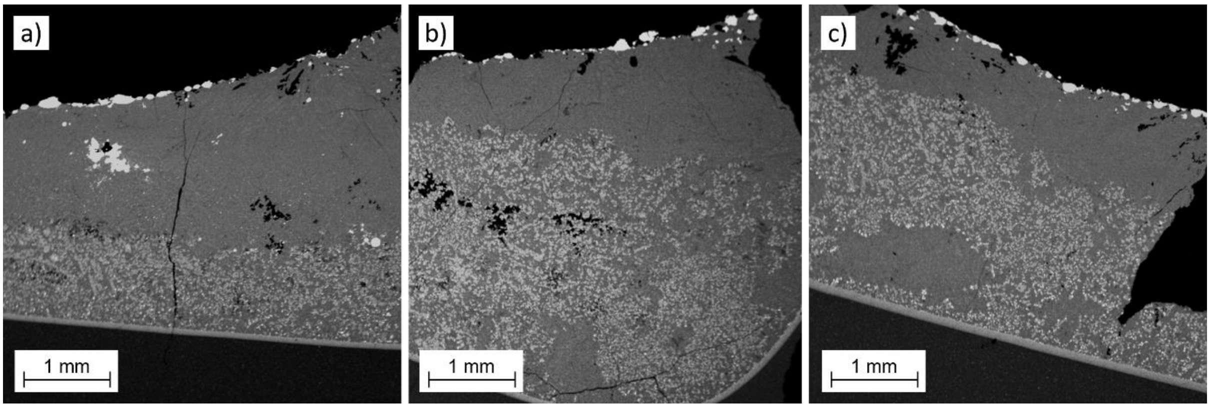

Figure S3. SEM BSE micrographs of (a) 20, (b) 30, and (c) 40 min reductions with a 50:50 mixture of biochar and coke in experimental series 1.

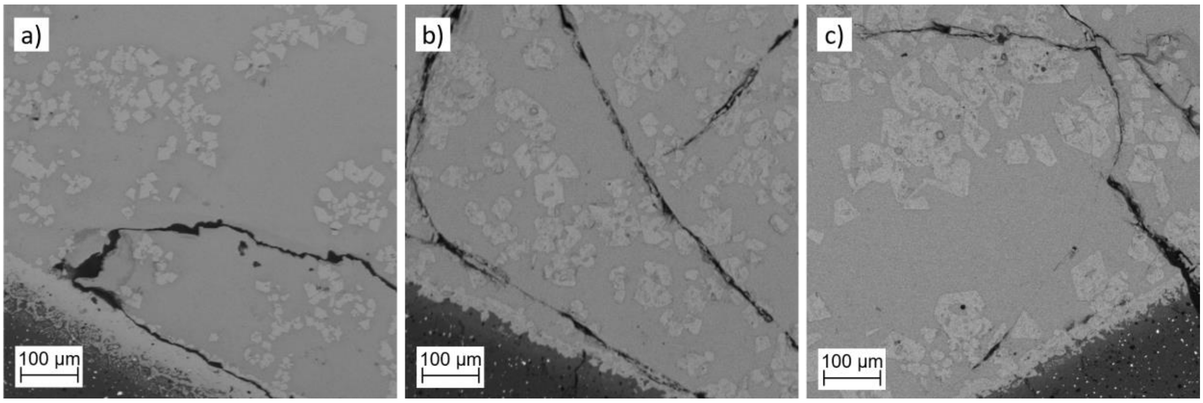

Figure S4. SEM BSE micrographs after the oxidation stage: (a) ES1, (b) ES2 (5 wt % MgO), 32 mL/min O2, (c) ES2 (5 wt % MgO), 65 mL/min O2.

Table S2. Slag basicity after all ES1 experiments, calculated as (CaO wt %+ MgO wt %) / SiO_2 wt %, based on SEM-EDS analysis.

		oxidation stage	20 min reduction	30 min reduction	40 min reduction
		0.73	-	-	-
reductant type	coke 100%	-	0.79	0.79	0.82
	biochar 100%	-	0.91	0.93	0.89
	coke 50%, biochar 50%	-	0.82	0.81	0.80

Table S3. Slag basicity after all ES2 experiments, calculated as (CaO wt % + MgO wt %) / SiO_2 wt %.

		oxidation stage		10 min reduction		30 min reduction		60 min reduction	
O_2 flow rate (mL/min)		32	65	32	65	32	65	32	65
MgO add. (wt %)	0	0.93	0.84	0.82	1.01	1.00	1.01	1.00	1.04
	5	0.96	0.97	0.99	1.03	0.99	0.91	1.04	1.04
	10	0.95	1.13	1.01	1.05	1.00	1.13	0.98	1.07
	15	-	-	1.11	1.11	-	-	-	-

Possible reactions and reaction enthalpies during the reduction of the iron oxides[1,2,3] are presented in equations (S1)–(S7):

$$3Fe_2O_3 + CO \rightarrow 2Fe_3O_4 + CO_2, \quad \Delta^\circ_{298}H = -39.6 \text{ kJ/mol} \quad (S1)$$

$$Fe_3O_4 + 4CO \rightarrow 3Fe + 4CO_2, \quad \Delta^\circ_{298}H = -16.3 \text{ kJ/mol} \quad (S2)$$

$$Fe_3O_4 + CO \rightarrow 3FeO + CO_2, \quad \Delta^\circ_{298}H = 37.4 \text{ kJ/mol} \quad (S3)$$

$$FeO + CO \rightarrow Fe + CO_2, \quad \Delta^\circ_{298}H = -17.9 \text{ kJ/mol} \quad (S4)$$

$$3Fe_2O_3 + C \rightarrow 2Fe_3O_4 + CO, \quad \Delta^\circ_{298}H = 132.8 \text{ kJ/mol} \quad (S5)$$

$$3Fe_3O_4 + C \rightarrow 3FeO + CO, \quad \Delta^\circ_{298}H = 50.1 \text{ kJ/mol} \quad (S6)$$

$$2FeO + C \rightarrow Fe + CO, \quad \Delta^\circ_{298}H = 36.9 \text{ kJ/mol} \quad (S7)$$

The amount of reductant needed in the reduction experiments was calculated based on stoichiometry, considering the fixed carbon content of the used reductant. For the calculations, it was assumed that during the reduction stage, Fe_2O_3, ZnO, PbO, and As_2O_5 are reduced according to the reactions (S8)–(S11):

$$Fe_2O_3 + C = 2FeO + CO \quad (S8)$$

$$ZnO + C = Zn + CO \quad (S9)$$

$$PbO + C = Pb + CO \quad (S10)$$

$$As_2O_5 + 5C = 2As + 5CO \quad (S11)$$

Reactions (8)–(11) take place in the molten slag and in a solution phase, and therefore their degrees of completion depend on the properties of the matrix phase, i.e. the slag or alloy.

References

(1) Chen, W.; Hsu, C.; Du, S. Thermodynamic Analysis of the Partial Oxidation of Coke Oven Gas for Indirect Reduction of Iron Oxides in a Blast Furnace. *Energy* **2015**, *86*, 758–771. DOI: 10.1016/j.energy.2015. 04.087.

(2) Liu, G.; Strezov, V.; Lucas, J.A.; Wibberley, L.J. Thermal Investigations of Direct Iron Ore Reduction with Coal. *Thermochim. Acta* **2004**, *410* (1), 133–140. DOI: 10.1016/S0040-6031(03)00398-8.

(3) Murakami T.; Kasai, E. Reduction Mechanism of Iron Oxide–Carbon Composite with Polyethylene at Lower Temperature. *ISIJ Int.* **2011**, *51* (1), 9–13. DOI: 10.2355/isijinternational.51.9.
